# Risk factors for poor outcome in community-onset *Clostridium difficile* infection

**DOI:** 10.1186/s13756-018-0365-6

**Published:** 2018-06-15

**Authors:** Eunyoung Lee, Kyoung-Ho Song, Ji Yun Bae, Doran Yoon, Joo-Hee Hwang, Pyoeng Gyun Choe, Wan Beom Park, Ji Hwan Bang, Eu Suk Kim, Sang Won Park, Nam Joong Kim, Myoung-don Oh, Hong Bin Kim

**Affiliations:** 1 Department of Internal Medicine, Seoul National University Bundang Hospital, 82 Gumi-ro 173 beon-gil, Bundang-gu, Seongnam, 463-707 Republic of Korea; 2 Department of Internal Medicine, Seoul National University College of Medicine, Seoul, Republic of Korea

**Keywords:** *Clostridium difficile*, Community-acquired infections, Anemia, Proton pump inhibitors, Mortality, Risk factors

## Abstract

**Background:**

A substantial portion of *Clostridium difficile* infection (CDI) cases occur in communities, and community-onset CDI (CO-CDI) can lead to serious complications including mortality. This study aimed to identify the risk factors for a poor outcome in CO-CDI.

**Methods:**

We performed a retrospective review of all inpatients with CDI, in a 1300-bed tertiary-care hospital in Korea, from 2008 through 2015. CO-CDI was defined as CDI occurring within 48 h of admission. Poor outcome was defined as follows: 1) all-cause 30-day mortality, 2) in-hospital mortality, or 3) surgery due to CDI.

**Results:**

Of a total 1256 CDIs occurring over 8 years, 152 (12.1%) cases were classified as CO-CDI and 23 (15.1%) had a poor outcome, including 22 (14.5%) cases of mortality and 2 (1.3%) cases of surgery. Patients with a poor outcome had a higher mean age than those without a poor outcome (75.8 vs. 69.6 years, *p* = 0.03). The proportion of men and prior proton pump inhibitor (PPI) use were significantly higher in the poor outcome group (65.2% vs. 41.9%, *p* = 0.04; 39.1% vs. 17.6%, *p* = 0.02, respectively). Multivariate binary logistic model showed that PPI use and anemia (hemoglobin < 10 g/dL) at presentation were significantly associated with a poor outcome (adjusted odds ratio [aOR], 3.76; 95% confidence interval [95CI], 1.26–11.21, aOR, 4.67; 95CI, 1.52–14.34, respectively).

**Conclusions:**

Clinicians should not only be aware of the possibility of CDI in the community setting but also pay more attention to PPI-using elderly patients with anemia in consideration of a poor outcome.

**Electronic supplementary material:**

The online version of this article (10.1186/s13756-018-0365-6) contains supplementary material, which is available to authorized users.

## Background

*Clostridium difficile* is an anaerobic spore-forming bacterium that is resistant to gastric acid; thus, it can stay in the intestinal tract and proliferate when the normal gut flora is disrupted under specific circumstances such as antibiotic agent usage. *C. difficile* infection (CDI) has been identified as a major cause of nosocomial diarrhea. CDI can show various manifestations, from mild diarrhea to severe colitis or toxic megacolon, which can result in surgery or even mortality.

The incidence of CDI has been increasing over the last 10 to 20 years, and several outbreaks of CDI have occurred globally [1]. In the United States, costs associated with CDI exceed $1.1 billion per year, even with conservative estimates [2]. Recent studies have reported that a substantial portion of CDI occurs in the community and its incidence has been increasing [3]. In a previous report, only 24.2% of CDI occurred during hospitalization in the United States [4]. An Australian study reported that community-onset CDI (CO-CDI) accounted for 29% of CDI [5]; in a Taiwanese report, CO-CDI accounted of 15% CDI when the percentage of community-acquired CDI and community-onset healthcare-associated CDI were combined [6]. Moreover, some CO-CDI resulted in poor outcomes in those reports [5, 6].

Age over 70 years, leukocytosis, hypoalbuminemia, increased creatinine, nasogastric tube feeding, and small bowel obstruction or ileus are known risk factors for severe CDI and are associated with a high mortality [7, 8]. However, some reports have revealed that patients with CO-CDI show different characteristics compared to patients with traditional nosocomial infection [9]. It is possible that the manifestations of complicated CO-CDI differ from those of nosocomial infection; however, few studies have investigated the risk factors for complications in CO-CDI. Thus, this study aimed to evaluate the clinical characteristics of CO-CDI and to identify the risk factors for a poor outcome in CO-CDI.

## Methods

### Study design and definitions

We performed a retrospective medical review in Seoul National University Bundang Hospital, which is a 1300-bed tertiary-care hospital located in Seongnam, Gyeonggi-do, South Korea. A medical record review was performed for all adults (≥ 18 years old) admitted from 2008 to 2015.

CDI was defined as a positive *C. difficile* toxin test and diarrhea or an equivocal *C. difficile* toxin test, diarrhea, and treatment for CDI. Diarrhea was defined as described in the medical records or as defecation exceeding 3 times in a day. ELISA was used to identify *C. difficile* toxin A or B (Gemini analyser, Stratec Biomedical GmbH, Birkenfeld, Germany), performed as per the manufacturer’s instructions in a single microbiology laboratory. CO-CDI was defined as CDI occurring within 48 h of admission. We regarded patients who were recurrently admitted multiple times within 1 week as one episode on the first admission day. When CDI occurred more than two times during one admission, we regarded the latter case as nosocomial CDI, which was excluded from this review. Poor outcome was defined if one of the following events occurred: 1) all-cause 30-day mortality, 2) in-hospital mortality or 3) surgery due to CDI. CDI treatment was defined as the use of intravenous/oral metronidazole, oral vancomycin, or both.

### Study measures

We collected epidemiological information such as sex, age, admission date, medical history including cardiovascular disease, diabetes mellitus, renal disease, hepatic disease, malignancy, and previous CDI from the medical records. A recent event of previous CDI was defined as an event occurring within the most recent 3 months. We reviewed recent medication histories such as antibiotic agent and proton pump inhibitor (PPI) use for the previous 1 month. We collected data on clinical manifestations (vital signs such as blood pressure, body temperature, Glasgow coma scale, stool frequency > 10 per day, presence of mucoid stool, duration of diarrhea before admission, abdominal pain, abdominal tenderness, intensive care unit [ICU] admission), laboratory values (white blood cell [WBC] count, hemoglobin, creatinine, albumin, glucose), radiographic data (plain radiography, computed tomography [CT]), and endoscopic results. Duration from admission to the treatment of CDI in days and the number of patients who needed to continue possible offending antimicrobial agents were reviewed. Laboratory data within 24 h from admission were collected and values with the greatest deviations from normal were collected if multiple values were available. In addition, medical images taken within 7 days of the *C. difficile* toxin test were reviewed.

### Statistical analysis

The independent Student *t*-test or Mann-Whitney *U*-test was used to compare continuous variables between the poor and good outcome groups, depending on the normality of data. The chi-square test was used for categorical variables. Linear regression was used to evaluate trends of incidence. To evaluate independent factors for a poor outcome in CO-CDI, we designed two logistic regression models. The first model included significant demographic and clinical variables identified from univariate analysis. The second model included significant laboratory variables which were identified from univariate analysis and are known to be associated with severity in previous studies. We separated the variables into two models rather than combining all of them into one model. This was because a small study population and a large number of variables and covariates with potential collinearity might have affected the power of the study and the statistical relevance of the results. Two-sided tests were performed and *P*-values of < 0.05 were considered statistically significant. The incidence rate was calculated for 10,000 admissions. All statistical analyses were performed with SAS for Windows, version 9.4 (SAS Institute, Inc., Cary, NC, USA).

## Results

### Characteristics of the population

During the 8-year study period, a total of 1256 CDI episodes were identified. Of these, 152 were classified as CO-CDI. Although the number of cases of CDI increased annually, the number of *C. difficile* toxin testing was also increased from 172 CDI cases/861 tests in 2008 to 256 CDI cases/2072 tests in 2015 (Additional file 1: Figure S1). However, there was no significant trend in the CDI incidence rate after the conversion 10,000 admission rates per month (*p* = 0.36, overall 32.75 per 10,000 admissions per month). There was no particular trend in the CO-CDI incidence rate (*p* = 0.51, overall 3.64 per 10,000 admissions per month). No significant trend was observed in the incidence rate of complicated CO-CDI (*p* = 0.84, overall 0.55 per 10,000 admissions per month).

The mean patient age was 70.66 (SD 14.89), with a slight female predominance (54.6%) (Additional file 1: Table S1). There were 37 (24.3%) cases from the long-term care facility. The mean Charlson’s comorbidity index was 2.09 (SD 1.90) and there were 23 (15.0%) cases with previous CDI; 119 (79.3%) cases had a history of antibiotic agent usage within the previous 1 month. Fifteen (9.9%) cases did not have an antibiotic or admission history for 3 months. Thirty-one (21.0%) cases had a history of prior PPI use. There were 42 (27.6%) cases with shock at the initial presentation. Eleven (7.2%) cases required ICU admission.

We identified the prior use of antibiotic agents within 1 month in 110 (71.9%) cases of CO-CDI. The antibiotic classification was ascertained in 95 (62.5%) cases. The most frequently used antibiotic agent was cephalosporin and it was used for 48 (50.5%) cases. Following, in the order of frequency, are fluoroquinolone in 37 (39.0%), β-lactam + β-lactamase inhibitor in 19 (20.0%), metronidazole in 9 (9.5%), carbapenem in 8 (8.4%) cases. Clindamycin was used in 2 (2.1%) cases.

### Risk factors for a poor outcome in CO-CDI

There were 23 (15.1%) cases with a poor outcome. Of these, there were 22 (14.5%) cases of mortality: 19 (12.5%) during the admission period and 14 (9.2%) within 30 days from admission. There were 2 cases of surgery, and of these, mortality occurred during admission in one case.

The mean age of patients and the proportion of men in the poor outcome group was higher than that in the good outcome group (75.8 vs. 69.6 years, *p* = 0.01; 65.2% vs. 41.9%, *p* = 0.04, respectively; Additional file 1: Table S1). The mean heart rate within 24 h after admission was significantly higher in the poor outcome group (109.80 vs. 98.1 beats per min, *p* = 0.01); however, there was no significant difference in the proportion of septic shock or mean body temperature between the two groups. The rate of prior PPI use was higher in the poor outcome group (39.1% vs. 17.6%, *p* = 0.02). The rate of prior antibiotic agent use was not significantly different between the two groups (73.9% vs. 80.3%, *p* = 0.49). When analyzed by the classification of possible offending antibiotic agents, penicillin derivatives were more frequently used in the poor outcome group (40.0% vs. 16.3%, *p* = 0.03; Additional file 1: Table S2). Clinical manifestations including mucoid stool, proportion of stool frequency exceeding 10 times, duration of symptoms, and abdominal pain or tenderness were not significantly different between the two groups. Laboratory values at presentation showed that the mean WBC count was higher in the poor outcome group, but this finding was not statistically significant. The mean hemoglobin level was marginally lower in the poor outcome group (10.42 g/dL vs. 11.30 g/dL, *p* = 0.06), and the mean bicarbonate and albumin levels were significantly lower in the poor outcome group than in the good outcome group (19.52 mmol/L vs. 21.84 mmol/L, *p* = 0.02; 2.63 g/dL vs. 3.21 g/dL, *p =* 0.0001, respectively).

Multivariate binary logistic analysis revealed that a history of PPI use was independently associated with a poor outcome (adjusted odds ratio [aOR], 3.88; 95% confidence interval [95CI], 1.310–11.518). In addition, anemia of hemoglobin under 10 g/dL was associated with a poor outcome (aOR, 3.55; 95CI, 1.230–10.219). Men were marginally associated with the increased risk of a poor outcome in CO-CDI (aOR, 2.74; 95CI, 0.998–7.525; Table 1).

**Table 1:** Risk factors for poor outcome in community-onset *Clostridium difficile* infection in multivariate logistic regression analyses

Variable	Odds ratio	95% CI	*P*
Age > 70 y	1.47	0.468–4.627	0.51
Male	2.74	0.998–7.525	0.05
Long-term care facility residence	0.98	0.287–3.354	0.98
Glasgow coma scale ≤12	2.06	0.589–7.185	0.26
Presentation with septic shock	0.84	0.256–2.742	0.77
Heart rate ≥ 100 /min	2.81	0.972–8.102	0.06
Prior proton pump inhibitor use within 1 month	3.88	1.310–11.518	0.01
ICU admission	2.71	0.599–12.257	0.20
Max leukocyte count > 20,000 /mL	2.78	0.897–8.684	0.08
Min hemoglobin level < 10 g/dL	3.55	1.230–10.219	0.02
Min bicarbonate level < 20 mmol/L	2.16	0.736–6.356	0.16
Max glucose level > 150 mg/dL	0.83	0.259–2.623	0.74
Max creatinine level > 2 mg/dL	0.54	0.160–1.841	0.33
Min albumin level < 2.5 g/dL	1.21	0.373–3.928	0.75

*ICU* Intensive care unit*, Max* maximum, *Min* Minimum, *CI,* confidence interval

Poor outcome was defined if at least one of following event was occurred: 1) all-cause 30-day mortality, 2) in-hospital mortality, or 3) surgery due to *C. difficile* infection

Clinical variables (n-152) and laboratory variables (*n* = 151) were analyzed in two separated models in consideration of potential collinearity

Laboratory results were the most deviated value from normal range within 24 h after admission

## Discussion

This study analyzed 152 CO-CDI episodes from a total of 1368 CDI cases, giving the proportion of 12.1%. A substantial proportion (15.1%) of CO-CDI cases underwent surgery and/or expired. Prior PPI use and anemia under hemoglobin 10 g/dL were associated poor outcomes in multivariate analyses.

The prevalence of CO-CDI has been reported to range between 10 and 42% in various countries [5, 6, 10]. We observed that CO-CDI comprised 12.1% of CDI and this result is comparable with these previous studies. In this study, in-hospital mortality and 30-day mortality were 12.5 and 9.2%, respectively. These rates are similar to findings in previous CO-CDI reports and are also comparable with the mortality in nosocomial CDI [6]. The factors associated with a poor outcome in CO-CDI were similar to those noted in previous studies in the univariate analysis but were slightly different in the multivariate analysis. Male sex and old age, similar to that in our study, have been identified as predictive risk factors for severe CDI in previous reports [7]. Elderly patients over 70 years comprised 62.5% of CO-CDI patients in our study. We confirmed that CO-CDI occurred more in the elderly and that they are more prone to have a poor outcome. In agreement with a previous report by Khanna et al. [10] which showed that CO-CDI in relatively young patients may follow a milder clinical course, we demonstrated that older patients with CO-CDI still had a higher risk of a poor outcome.

To the best of our knowledge, no previous report has attempted to clarify the risk factors for severe outcomes in CO-CDI. This study demonstrated that PPI use is associated with a poor outcome in CO-CDI, similar to that in nosocomial CDI. PPI is known to be associated with the development [9, 11], severity [12], and recurrence [8] of CDI, and has recently been reported to be associated with the development of CO-CDI [13]. In a pharmacoepidemiologic cohort study, Howell et al. [14] reported that iatrogenic gastric acid suppression increases CDI development with a dose-response effect, suggesting a causal relationship. In an in vitro study, Jump et al. [15] reported the prolonged survival of *C. difficile* in the gastric contents of PPI-using patients with elevated pH (pH > 5). Another study reported that exposure to PPI promoted toxin gene expression of *C. difficile* [16]. Furthermore, an experimental study with mice demonstrated that toxin A and B titers and myeloperoxidase activity, which is a marker for inflammatory activity was elevated in both antibiotic and proton pump inhibitor-receiving groups [17]. This finding suggests that PPIs should be appropriately used.

Anemia reflects the general condition of a patient, such as nutritional status (e.g. iron, vitamin deficiency), as well as underlying chronic diseases. However, we could not find any previous report regarding a direct association of anemia with human CDI. In a study, an association between anemia and colitis was noted in a mouse acute colitis model [18]. Anemia is also influenced by the pro-inflammatory cytokine-mediated inhibition of erythropoiesis [19]. It is possible that anemia in patients at the initial presentation of CDI might represent the severity of CDI associated with relative ischemia in an inflammatory condition of the colon. Thus, we thought that anemia could be a significant factor for poor outcomes in CO-CDI, as a representative of an underlying condition and severity of acute illness.

Regarding possible offending antibiotic agents, cephalosporin was used most frequently, followed by fluoroquinolone, and β-lactam + β-lactamase inhibitor in a descending order. Conventionally, clindamycin is a known risk factor for nosocomial CDI and CO-CDI [20]. In this report, only 2 patients were administered clindamycin before admission. One reason for this is that clinicians may have avoided prescribing clindamycin because of the associated risk of CDI. Additionally, broad-spectrum antibiotics such as β-lactam + β-lactamase inhibitor or carbapenem are being used increasingly, decreasing the need for extra anaerobic bacterial coverage [21]. Except for clindamycin, three dominant antibiotic classes in this study were noted among previously reported drugs associated with CO-CDI risk in a meta-analysis [22].

This study has some limitations. The first limitation of this study includes its single-center retrospective study design. Cases of CDI could have been missed if primary physicians did not suspect CDI and did not test for the *C. difficile* toxin in patients from the community with relatively mild diarrhea, who did not have a history of antibiotic use. Thus, the CDI prevalence rate could have been underestimated. This assumption might explain the older age and higher proportion of febrile patients in this study compared to a previous community-based study [23]. However, we believe that our study included sufficient cases of severe CO-CDI and is valuable in this regard. Second, we did not investigate whether microbiological characteristics (e.g., ribotype) are associated with complicated CO-CDI. In a nationwide study from 12 hospitals in Korea, a major strain of nosocomial CDI was ribotype 017 [24]. There was limited data on the *C. difficile* strain in the case of CO-CDI in Korea, and we did not find any report on the relationship between ribotype and severity. Although the hypervirulent strain of ribotype 027 was described for the first time in Korea in 2009 [25], it does not appear to be epidemic or endemic in Korea yet. Considering previous reports on the prevalence rate in Korea, it does not appear to be an epidemic of a hypervirulent strain based on the prevalence rate compared to a previous study in Korea [26].

## Conclusions

In this study, a substantial proportion of CDI occurred in the community. Clinicians should suspect CO-CDI in patients with diarrhea, abdominal pain, abdominal tenderness, and fever, who have a history of antibiotic use. In particular, patients with anemia at presentation and who use PPI need additional attention considering the identification of anemia and PPI use as risk factors for a poor outcome.

## Additional file

Additional file 1: **Table S1.** Clinical characteristics of patients with community-onset Clostridium difficile infection. **Table S2.** Possible offending antibiotic agents in patients with community-onset Clostridium difficile infection. **Figure S1.** Incidence of *C. difficile* infection from 2008 through 2015 (DOCX 183 kb)

## Abbreviations

95CI: 95% confidence interval
aOR: Adjusted odds ratio
CDI: *Clostridium difficile* infection
CO-CDI: Community-onset *Clostridium difficile* infection
CT: Computed tomography
PPI: Proton pump inhibitor
WBC: White blood cell
