# Supplementary material for: Risk factors for poor outcome in community-onset Clostridium difficile infection
Source: Antimicrob Resist Infect Control. 2018 Jun 15;7:75. doi: 10.1186/s13756-018-0365-6 (PMC6003152; doi:10.1186/s13756-018-0365-6)
Supplement: Supplementary file 1 — Table S1. Clinical characteristics of patients with community-onset Clostridium difficile infection. Table S2. Possible offending antibiotic agents in patients with community-onset Clostridium difficile infection. Figure S1. Incidence of C. difficile infection from 2008 through 2015 (DOCX 183 kb) [file 13756_2018_365_MOESM1_ESM.docx]

**Additional file**

**Risk factors for poor outcome in community-onset Clostridium difficile infection**

Eunyoung Lee, Kyoung-Ho Song, Ji Yun Bae, Doran Yoon, Joo-Hee Hwang, Pyoeng Gyun Choe, Wan Beom Park, Ji Hwan Bang, Eu Suk Kim, Sang Won Park, Nam Joong Kim, Myoung-don Oh, Hong Bin Kim

Contents:

Table S1. Clinical characteristics of patients with community-onset Clostridium difficile infection -------------------------------------------------------------------------------------- p2

Table S2. Possible offending antibiotic agents in patients with community-onset Clostridium difficile infection ----------------------------------------------------------------------------- p4

Figure S1. Incidence of C. difficile infection from 2008 through 2015 --------------- p5

**Table S1. Clinical characteristics of patients with community-onset *Clostridium difficile* infection**

| Characteristics | All (%) (n=153) | Poor outcome (n=23) | Good outcome (n=129) | *P* |
| --- | --- | --- | --- | --- |
| Sex (male) | 69 (45.39) | 15 (65.22) | 54 (41.86) | 0.04 |
| Age (years) | 70.66 ± 14.89 | 75.78 ± 10.80 | 69.57 ± 15.28 | 0.01 |
| Septic shock | 42 (27.63) | 9 (39.13) | 33 (25.58) | 0.18 |
| Heart rate (/min) | 99.85 ± 20.52 | 109.80 ± 21.82 | 98.07 ± 19.84 | 0.01 |
| GCS | 13. 44 ± 3.00 | 12.26 ± 3.51 | 13.65 ± 2.87 | 0.04 |
| Body temperature (°C) | 37.30 ± 0.93 | 37.40 ± 1.00 | 37.28 ± 0.92 | 0.61 |
| Comorbidity index | 2.09 ± 1.90 | 2.39 ± 1.47 | 2.03 ± 1.96 | 0.18 |
| Long-term care facility | 37 (24.34) | 8 (34.78) | 29 (22.48) | 0.21 |
| Previous CDI | 23 (15.03) | 5 (21.74) | 18 (13.95) | 0.34 |
| Prior antibiotic agent | 119 (79.33) | 17 (73.91) | 102 (80.31) | 0.49 |
| Prior PPI | 31 (20.95) | 9 (39.13) | 22 (17.60) | 0.02 |
| Mucoid stool | 20 (13.16) | 2 (8.70) | 18 (13.95) | 0.49 |
| Frequency>10/day | 18 (11.84) | 4 (17.39) | 14 (10.85) | 0.37 |
| Symptom duration (days) | 6.40 ± 9.97 | 5.83 ± 8.97 | 6.50 ± 10.17 | 0.61 |
| Abdominal pain | 69 (45.39) | 11 (47.83) | 58 (44.96) | 0.80 |
| Abdominal tenderness | 67 (44.08) | 11 (47.83) | 56 (43.41) | 0.69 |
| Ileus (n=136) | 69 (51.11) | 8 (38.10) | 61 (53.51) | 0.19 |
| abnormal CT (n=84) | 53 (63.1) | 21 (80.77) | 32 (55.17) | 0.20 |
| endoscopic PMC (n=49) | 38 (77.55) | 14 (100) | 24 (68.57) | 0.18 |
| White blood count (/mL) | 15606.51 ± 11573.34 | 19493.9 ± 14653.5 | 14913.4 ± 10858.4 | 0.10 |
| Hemoglobin (g/dL) | 11.16 ± 2.06 | 10.42 ± 2.57 | 11.30 ± 1.94 | 0.06 |
| Bicarbonate (mmol/L ) | 21.49 ± 4.58 | 19.52 ± 3.60 | 21.84 ± 4.66 | 0.02 |
| Glucose (mg/dL) | 132.34 ± 52.04 | 132.7 ± 42.91 | 132.3 ± 53.67 | 0.34 |
| Creatinine (mg/dL) | 1.72 ± 1.64 | 1.59 ± 1.02 | 1.74 ± 1.73 | 0.62 |
| Albumin (g/dL) | 3.13 ± 0.68 | 2.63 ± 0.55 | 3.21 ± 0.66 | 0.0001 |
| ICU admission | 11 (7.24) | 4 (17.39) | 7 (5.43) | 0.04 |
| Treatment start day | 1.69 ± 2.29 | 1.74 ± 2.65 | 1.68 ± 2.22 | 0.56 |
| No. of continuing offending agents (n=95) | 12 (12.63) | 3 (20.00) | 9 (11.25) | 0.35 |

GCS, Glasgow coma scale; CDI, Clostridium difficile infection; PPI, Proton pump inhibitor; CT, computed tomography; PMC, pseudomembranous colitis; ICU, Intensive care unit

Poor outcome was defined if at least one of following event was occurred: 1) all-cause 30-day mortality, 2) in-hospital mortality, or 3) surgery due to *C. difficile* infection.

Values are expressed as mean ± standard deviation or subjects number (%).

**Table S2. Possible offending antibiotic agents in patients with community-onset *Clostridium difficile* infection**

| Antibiotics | Poor outcome (n=15) | Good outcome (n=80) | *P* |
| --- | --- | --- | --- |
| Cephalosporin (%) | 8 (53.33) | 40 (50.00) | 0.75 |
| Fluoroquinolone (%) | 6 (40.00) | 31 (38.75) | 0.86 |
| Glycopeptide (%) | 1 (6.67) | 4 (5.00) | 0.77 |
| Metronidazole (%) | 3 (20.00) | 6 (7.50) | 0.12 |
| β-lactam + β-lactamase inhibitor (%) | 6 (40.00) | 13 (16.25) | 0.03 |
| Macrolide (%) | 1 (6.67) | 2 (2.50) | 0.38 |
| Carbapenem (%) | 1 (6.67) | 7 (8.75) | 0.82 |
| Trimethoprim-sulfamethoxazole (%) | 1 (6.67) | 3 (3.75) | 0.59 |
| Others (%) | 0 | 8 (10.00) | - |

Poor outcome was defined if at least one of following event was occurred: 1) all-cause 30-day mortality, 2) in-hospital mortality, or 3) surgery due to *C. difficile* infection.

Others included 2 cases with anti-tuberculosis drugs, 2 with clindamycin, 2 with aminoglycoside, and 2 with primaquine.

**Figure legend**

**Fig. S1.** Incidence of *C. difficile* infection from 2008 through 2015. The number of *C. difficile* toxin tests increased annually (A). However, no statistically significant trend was shown after converting to 10,000 admission rates per month for the total *C. difficile* infection (B), community-onset *C. difficile* infection (C), and complicated community-onset *C. difficile* infection (D) during the study period.


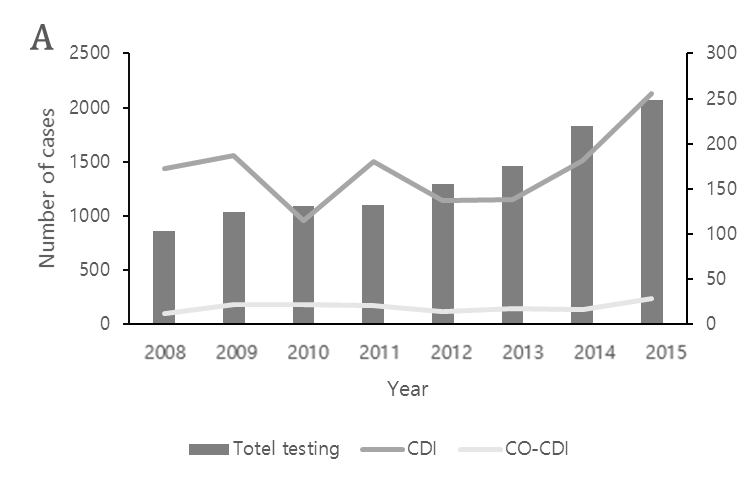


***Fig. S1. A***


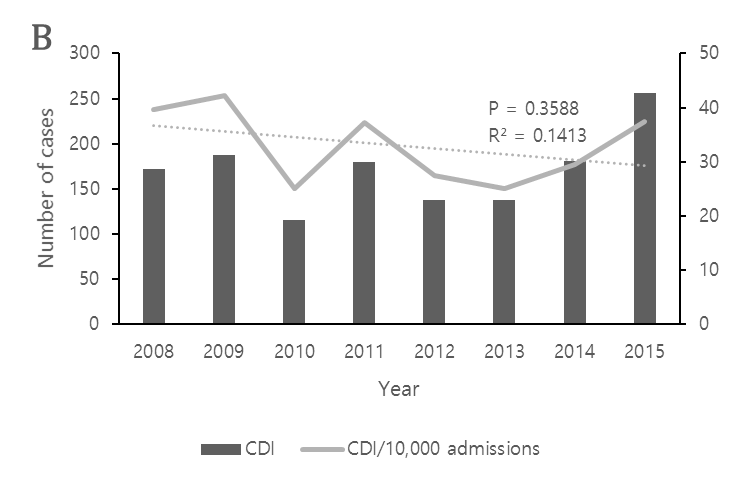


***Fig. S1. B***


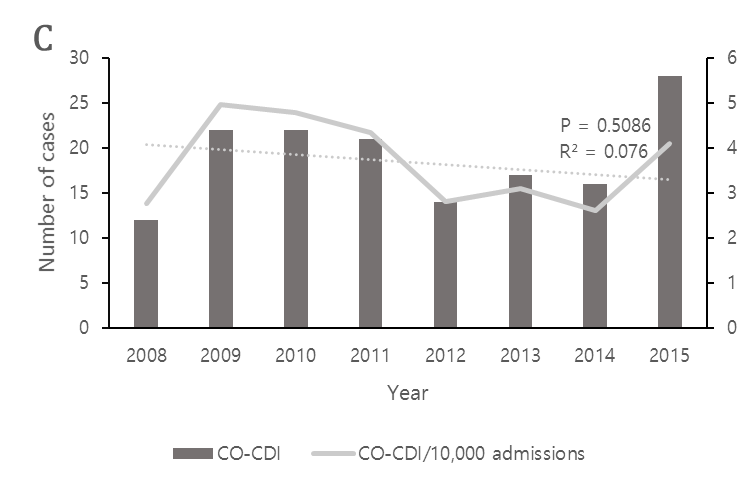


***Fig. S1. C***


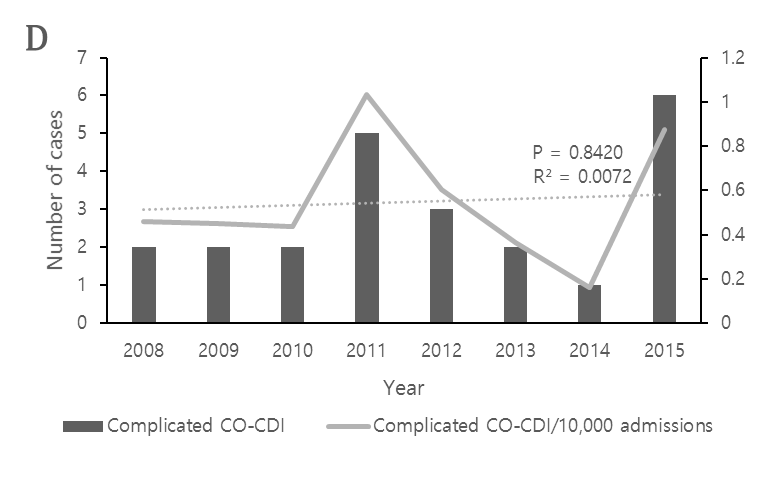


***Fig. S1. D***
